# Child health care nurses' experience of language screening for 2.5‐year‐old children: A qualitative study

**DOI:** 10.1002/nop2.1918

**Published:** 2023-06-14

**Authors:** Anna Lindgren Fändriks, Kjerstin Almqvist, Fredrik Hjärthag, Karin Pernebo

**Affiliations:** ^1^ Department of Social and Psychological Sciences Karlstad University Karlstad Sweden; ^2^ Department of Research and Development Region Värmland Karlstad Sweden; ^3^ Department of Psychology Linnaeus University Växjö Sweden; ^4^ Department of Research and Development Region Kronoberg Växjö Sweden

**Keywords:** child health care, early language screening, qualitative methods

## Abstract

**Aim:**

To investigate the experience of Child Health Care Nurses (CHCNs) using language screening for 2.5‐year‐old children.

**Design:**

An exploratory qualitative design with an inductive approach.

**Method:**

Data were collected through semi‐structured, interviews with Swedish CHCNs who regularly performed language screening for children. The interviews were analysed by thematic analysis.

**Results:**

Four themes were identified: ‘The difficult visit’, ‘Explanations for language delay’, ‘Language screening across cultures’ and ‘Language screening with children exposed to adverse life events’.

**Patient or Public Contribution:**

Our findings suggest that in routine care a modified procedure is used for the language screening of children aged 2.5 to secure the child's cooperation and to preserve an alliance with the parents. Consequently, the validity of the screening is called into question, particularly when it comes to children from families with origins outside the dominant culture and children exposed to adverse life events.

## 1 INTRODUCTION

Most children's language acquisition occurs within a predictable time frame. Although the phenomenon is highly robust, some children exhibit deviations in language development. Approximately, 5%–8% of the child population exhibit various language delays (Law, Charlton, & Asmussen, 2017). In a Swedish population‐based study the prevalence of language delays for children was 6% (Miniscalco, 2003). Variances in reported prevalence is probably mainly due to differences in terminology (Bishop, 2017). A child's early environment, genetic predisposition, interaction with caregivers, risk factors such as neglect or exposure to violence, and socio‐economic conditions, all influence language development (Bruce et al., 2022; Di Sante et al., 2020; Lum et al., 2018; Rowe & Weisleder, 2020). Children's early language development is of interest for several reasons. It is one of the most powerful predictors of the child's learning to read and succeeding in school (Hayiou‐Thomas et al., 2017; Law, Levickis, et al., 2017). It has also been associated with: cognitive impairments and neuropsychiatric disorders emotional, behavioural and social functioning and adult mental health (Eadie et al., 2018; Law, Charlton, & Asmussen, 2017; Miniscalco et al., 2006; Schoon et al., 2010). Studies have repeatedly found that maltreated children have delayed language development and poorer social skills (Lum et al., 2018). There are several possible reasons for the association between maltreatment and poor language skills. Stress as a consequence of maltreatment is known to be a risk factor in neurobiological development, and maltreatment and other adverse events in childhood have been shown to affect cognitive development and mental health during childhood and adulthood (Lippard & Nemeroff, 2020; Nelson & Gabard‐Durnam, 2020). Early recognition of language delay and facilitation of appropriate interventions is thus an urgent task for child health care (CHC) (Law & Levickis, 2018).

## 2 BACKGROUND

The feasibility of universal screening for language delay in children has been questioned on the basis of limitations in sensitivity and specificity found in available screenings instruments (Jullien, 2021; Wallace et al., 2015). Wallace et al. (2015) concluded in a systematic review that there is not enough evidence of the effectiveness of general language screening for it to be recommended for implementation in primary health care settings (sensitivity 50%–94%, specificity 45%–96%). The results by Jullien (2021) are in line with earlier reviews (Voigt & Accardo, 2015; Wallace et al., 2015), concluding that there is insufficient evidence that language screening improves language outcomes. Additionally, language screening tests have been reported to not function as well in routine clinical work as in validation studies (Voigt & Accardo, 2015). Although some studies have shown promising results in support of universal screening (Holzinger et al., 2022; Kas et al., 2022), the use of universal language screening has not been recommended in primary care in many countries due to insufficient support of effectiveness (Jullien, 2021; Wallace et al., 2015).

In 1991 the Swedish National Board of Health and Welfare recommended structured language screening to be offered to all children 2.5–3 years old through the nationwide voluntary CHC. The CHC offers a publicly funded programme to all families with children up to the age of 6. The primary objectives of the CHC are to conduct surveillance of children's general health and development. The programme reaches almost every child; 99% of Swedish children attend the programme (Wallby & Hjern, 2011). The programme offers children a health visit, with a focus on language development at age 2.5–3. The assessment is performed by a Child Health Care Nurse (CHCN) to identify children with severe language and communication difficulties (Rikshandboken, 2022). An evaluation showed promising results for sensitivity and specificity with an acceptable rate of false positives and very few false negatives (Mattsson et al., 2001). A follow‐up study (2003) showed that children who had been identified at the 2.5‐year screening were at high risk of having persistent language problems at the age of 6 (Miniscalco, 2003). Furthermore, 62% of the children who had persistent language delays at age 6 had received a neurodevelopmental diagnosis such as autism, attention deficit hyperactivity disorder, or a combination of these (Miniscalco et al., 2006). Additionally, a Swedish study from 2021 found that parental concern alone was insufficient for identifying children with language delays (Nayeb et al., 2021).

The language screening for children aged 2.5 years has now been part of the Swedish national CHC programme for 20 years (Rikshandboken, 2022). The CHCNs play a crucial role in the language screening, yet to our knowledge there has been no study of the CHCN's experiences of performing the screening. The aim of this study was to investigate the CHCNs' experiences of using the language screening at 2.5 years.

## 3 METHODS

### 3.1 Design

A qualitative design was chosen with semi‐structured interviews analysed by thematic analysis. This methodology was chosen to explore CHCN's experiences of the language screening for children aged 2.5. The consolidated criteria for reporting qualitative research was used producing the manuscript (Tong et al., 2007).

### 3.2 Sample and setting

Interviews were conducted with 16 female nurses from five out of 31 child health care centres (CHCCs) in the county of Värmland in Sweden. Participants were recruited through purposive sampling. Sixteen out of 17 invited CHCNs agreed to participate. All were female and of Swedish origin with Swedish as their mother tongue. They regularly performed language development screening and had a minimum of 1 year experience using the screening for children aged 2.5. They were between 32 and 66 years old (*M* = 51 years, SD = 9,6 years) and had worked in the CHC for 2–28 years (*M* = 11 years, SD = 7,6 years). They were all specialist nurses; two in paediatric nursing and 14 in public health nursing. They worked in CHCs in urban and rural areas and the interviews were held in natural settings. Of the total, 40% worked in urban and 60% in rural areas.

### 3.3 Data collection

Individual semi‐structured interviews were conducted face‐to‐face, by the first author. The researcher who conducted the interviews was a Swedish, 34‐year‐old, female PhD student, who also works part time as lic. Psychologist in the CHC. The research leader had through previous research an established collaboration with the CHCs. The interviews took place in privacy at each nurse's office at her CHCC during October 2020 to February 2021. Interviews lasted from 40 to 75 minutes. The interviews were audio recorded and transcribed by the first author. Small changes were made following the initial interviews to ensure that information was collected on how the language screening was used. The researcher's interpretations and reflections were continuously discussed within the researcher group as the data were collected. The interviews focused on core questions about their experience of working with the language screening. The questions were open ended, for example: *How would you describe your experience of working with the language screening test for children aged 2.5? Could you describe the latest/most recent language development screening test you performed?* Probes and follow‐up questions were used to broaden or deepen the answers, for example: *Can you tell me more about that? Can you explain more?*

### 3.4 The language screening

The language screening is based on a parental questionnaire and an assessment performed by the CHCN. The CHCN observes and communicates with the child following a structured schedule. The CHCN uses a protocol to note if the child understands words and sentences, can imitate and name, and can use two‐word‐sentences. The CHCN also assesses the willingness of the child to communicate and interact verbally and non‐verbally. Parents are asked to fill out a questionnaire to assess the development of language and communication in their child. The questionnaire contains questions about the child's vocabulary, verbal comprehension, multiword utterances, use of gestures, oral motor skills and whether or not caregivers are worried about the child's language development. The final evaluation is made by combining the assessment of the nurse and that of the parents (Rikshandboken, 2022). If the child's language development is judged to be delayed the nurse can schedule a follow‐up visit or refer the child to a speech and language therapist or a psychologist.

### 3.5 Data analysis

The interviews were analysed using thematic analysis as described by Braun and Clarke (2006, 2019). The analysis was performed inductively. The process was guided by the research aim of identifying, analysing and reporting the CHCNs' statements about their experiences with the language screening. The present analysis was conducted in an essentialist/realist framework and themes were identified at a semantic level (Clarke & Braun, 2017).

**FIGURE 1:**
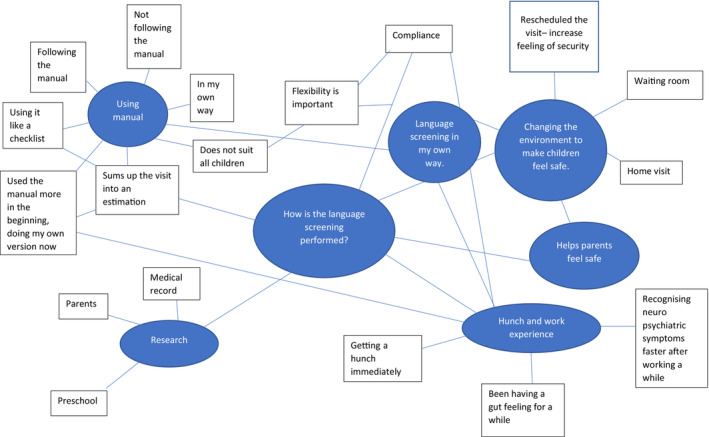
Example of mindmap early in the process for theme 1.

The analysis began with familiarisation to get to know the data by reading the full transcripts several times, following Braun and Clarke (2006, 2019). Then the data were systematically coded, with codes kept as close as possible to the wording used by the CHCNs to ensure that the data were representative of their statements. Two interviews were coded separately by three of the authors and the codes were compared to determine similarities and differences. When some minor differences were found, these were discussed by the authors. During the analysing process, the authors’ experience of qualitative research was weighed in as well as the first author's insight in CHC. In the dialogue various interpretations deepened the understanding of the statements and consensus could be reached. Codes were grouped within and across interviews using mind maps to generate initial themes (for an example of the mind maps see Figure 1). Main themes and subthemes were then checked against the original transcripts, and adjustments were made where needed. A narrative description of each theme was generated and given a title to capture the story the theme was telling. Quotes from the interviews connected to the codes were then chosen to represent the story of the theme. Each step was first carried out independently by the first author and then discussed and revised in collaboration with all authors. The themes and quotes were presented to the participants through a digital group seminar, where all participants were invited. During the seminar some of the participants provided verbal feedback and reflections. Almost all of the feedback was confirmatory, and stressed the importance of the results, some feedback resulted in deepened understanding of the themes and minor changes in the quotes. For example, some stressed the importance of a flexible approach to meet the needs of different families during the visits at the CHC.

### 3.6 Ethical considerations

First, written information about the study was given in a local CHCC information letter reaching all the CHC employees in Värmland. Second, five CHCCs, representative of areas with varying socio‐demographic backgrounds, were visited and information about the aim of the study was given orally. CHCNs at these units were contacted by email and 16 out of 17 CHCNs agreed to participate. The participants received verbal and written information before they were asked for informed written consent to participate. Each participant was given a unique code number and identifying information was removed from their transcript. The study was approved by REDACTED.

## 4 RESULTS

During the analytic process, four themes and eight subthemes were identified (Table 1). The themes are presented and illustrated by quotes from the CHCNs. Minor changes have been made in the wording to protect the CHCNs' integrity and to adjust for the translation from Swedish.

**TABLE 1:** Main themes and sub‐themes.

Main themes	Sub‐themes
The difficult visit	A modified screening procedure
	Parents' resistance
Explanations of language delay	Deficient language stimulation
	Neurodevelopmental disorders
Language screening cross cultures	Assessment of bilingual children
	Language stimulation in ethnic minorities
Language screening with children exposed to adverse life events	Language delay associated with exposure to adverse life events
	Trying to contribute to language development for exposed children

### 4.1 The difficult visit

At the beginning of most interviews the CHCNs stated that they experienced the visit at 2.5 years to be the most challenging visit in the national health programme. From the challenges identified, two sub‐themes emerged: *A modified screening procedure* and *parents' resistance*.

#### 4.1.1 A modified screening procedure

In the CHCNs' opinion, individual differences in child development were larger at age 2.5 than at other ages. Children were frequently shy and withdrawn at the beginning of the visit. Under the national health programme, a child's previous visit at the CHCC was 1 year earlier, at the age of 1.5. The CHCNs thought this gap might be an explanation for children being more shy and less likely to talk and participate.

They enter the door, and they haven't seen me in a year and they are supposed to talk to me and play with me, when they don't know who I am. I understand why they are sceptical, some of them, I do.

Difficulties in getting the children to participate and the large individual differences in development and personality required the CHCNs to modify the screening procedure to be able to assess the child's language ability. Some CHCNs followed the manual for the language screening assessment, but others created their own version of the screening procedure and used the manual more as a checklist. Some CHCNs reduced their use of the manual as they grew more experienced. One commented as follows: ‘We [CHCNs] have talked, and we know we don't do the same procedure. So, I think, yeah, we do it differently, I think, because of our different backgrounds and experience’.

Most of the CHCNs reported that they eavesdropped on the waiting room for an opportunity to hear the children speak. For some, when they could hear children speaking in the waiting room, they already knew that they would pass the screening. Some said that it was not only the procedure that differed between the CHCNs, but also the actual assessments themselves.

We do things entirely differently. I don't know why that is; maybe we are taught differently, or maybe the routines at the workplace are different … I have met 4‐year‐old children who did not have language skills at age 2.5 and nothing had been done at the time. And according to the parents the nurse had told them that it would pass and language development would start by itself.

#### 4.1.2 Parents' resistance

The CHCNs reported that some parents were prepared before the visit, bringing forms already filled in. Other parents said that they had not thought about their child's language development, were not interested in any advice, and did not accept referrals to a speech therapist or psychologist. ‘Some parents are excellent; they are well informed and maybe quite worried when they arrive … others have not thought about it at all. This might be the first time that they ever thought about it’. During the visit parents were also asked if they are worried about their child's language development. Sometimes it was difficult for the CHCNs to trust the information from the parents. When a CHCN felt uncertain they sometimes had to rely on the parents' judgement.

According to the parents the child can talk, but they might not have said a word in here, no matter how much I try to put the child at ease. I try to eavesdrop on the waiting room, If the child gets shy in here, at least I have heard that they talk, so I know if the parents are right or not. But sometimes I wonder: do they really talk in 2‐ or 3‐word sentences? ‘Yes, they do’ the parents say.

The CHCNs reported that one possible reason for their not always trusting the parents' assessments was the parents' desire for their children to be normal. A parent might feel ashamed of the child's disability or hope it would pass, or lack the ability to understand the child's needs. These circumstances could be a challenge for the CHCNs because they needed parental consent to help the children. ‘Some parents don't have the capacity, or maybe they are able understand their child at home. … So, it's important to encourage the parents to continue practicing speech’. Some parents were not interested in a referral to speech therapy or in signing up for a course about interventions for stimulating language. ‘Unfortunately, not many take the opportunity, at least not here, to join a class to show their support and for the information they could get there’.

### 4.2 Explanations of language delay

The CHCNs had various explanations for delayed language development in children, but two arose most frequently: *Deficient language stimulation* and *Neurodevelopmental disorders*.

#### 4.2.1 Deficient language stimulation

The CHCNs argued that many children with a language delay or poor vocabulary had experienced insufficient language stimulation at home. Language stimulation was described as parents talking, singing, reading and playing with their children. In their advice to parents the CHCNs emphasised how crucially important language stimulation was. They could see huge differences between children whose parents had stimulated language development and those whose parents had not.

You can see the difference in children who have been spoken to a lot when you do the language screening. At age 2.5 they know so much more, compared to those who come from a quiet home, who know a lot less.

#### 4.2.2 Neurodevelopmental disorders

For some children showing a language delay, the CHCNs suspected a neurodevelopmental disorder, primarily autism. Most CHCNs reported that they always looked for features of autism such as lack of eye contact or difficulty concentrating as part of the language screening. If the child did not make eye contact, did not follow probes, or behaved in a manner considered inappropriate, the CHCNs suspected autism. ‘If they are disruptive in here and do not talk, then I think it's autism’. Some CHCNs stated that if the family seemed well‐functioning and the parents had high expectations for the child's wellbeing, then neurodevelopmental disorders became an even more likely explanation of the child's delayed development. For some CHCNs their focus on evaluating patterns of autism might raise a risk of their suppressing other explanations, patterns or needs the child might have. ‘It's autism that I think of … and I think of that so much I have to stop myself, because … it doesn't have to be autism, it can be something else’.

### 4.3 Language screening cross cultures

All CHCNs described experiences with families from ethnic minorities. Some of the CHCNs worked in areas with a high percentage of the child population having non‐European immigrant parents. This theme included two sub‐themes: *Assessment of bilingual children* and *Language stimulation in ethnic minorities*.

#### 4.3.1 Assessment of bilingual children

When bilingual children were assessed, CHCNs used an interpreter by telephone. They identified several obstacles regarding how to evaluate the results from these screenings. ‘I think it's really hard with those who don't speak Swedish, because they often speak in their language and the parents say “Yes, that means car”’. The CHCNs had to modify the way they talked and communicated with the children and sometimes doubted the correctness of their evaluation. ‘You must trust what the parents say, because you can't decide by yourself, most of the time, how the language use works. It's really difficult’. A few CHCNs even reported that they knew they had made mistakes because of problems with translation.

#### 4.3.2 Language stimulation in ethnic minorities

The CHCNs described some differences arising from cultural issues in how parents understood and followed their advice. The CHCNs observed cultural differences in how parents stimulate language. There were also differences in the parents' attitudes towards children's language development, which could affect the parents' attitudes towards referral for the child. ‘Most of the parents who don't socialise or talk with their children that we meet here, come from an ethnic minority … I think it's because of lack of knowledge’. The CHCNs gave advice to parents from ethnic minorities on how to play with their children, how their children should and should not play with other children, and how to socialise to stimulate language development. Some CHCNs found the advice to have been effective: ‘the mother thought it was great; she would never have played with her children if the speech therapist hadn't shown her how to do it’.

Sometimes for a child with a language delay who was not fluent in Swedish or who spoke one or two different languages at home, the CHCN would advise a focus on one language. If children focused on one language, some CHCNs said, they might learn it properly, which would increase the likelihood of their making friends and facilitate future success in school. ‘We discuss that above all it can be good to learn one language, and learn it properly, instead of mixing four’.

### 4.4 Language screening with children exposed to adverse life events

The CHCNs described meeting children who had experienced neglect from their parents or other adverse life events, these statements resulted in two subthemes: *Language delay associated with exposure to adverse life events* and *Trying to contribute to language development for exposed children*.

#### 4.4.1 Language delay associated with exposure to adverse life events

The CHCNs described a connection between child experiences of neglect or exposure to adverse life events and delayed language development. They were aware of the parents' problems in these cases, for example, drug abuse, psychiatric illness, cognitive impairment, destructive conflicts between the parents, or domestic violence. ‘And we have some kids, where … language not quite started as expected. And that's post‐traumatic, the child psychiatrist thinks’. One possible explanation given for the connection between adverse life experiences and delayed language development was the parents' lack of language stimulation, arising from the context of the adverse life events. Parents with severe challenges had not had the time, capacity, or ability to read, sing, or spend time talking to their children.

I have a boy now who is coming for the 3‐year visit on Monday. A referral has been sent to the speech therapist, and the father is now alone with the 3‐year‐old and his younger siblings, and the mother is in drug‐abuse rehab. And, well, I don't think that he finds the time to read and things like that.

#### 4.4.2 Trying to contribute to language development for exposed children

The CHCNs described their struggle to motivate parents to stimulate language development for children exposed to adverse life events. They suggested reading, singing, talking, activities such as book bags from the library or they set a time for an appointment with a speech therapist. They worried about children with parents that did not make the effort required to help their children. ‘I think of the ones with no support at home … I mean, there is no commitment and interest in the child's delayed language development, compared to a family who is like “ha, we can do this”’. Some children with protected personal data or who were in foster care did not get a referral to a speech therapist, medical care or psychological help because of the consent needed from custodians. According the CHCNs all custodians had to give their consent for referrals, and sometimes it could be hard to reach them because of active drug abuse, or because one parent was afraid to be found by the other parent. Sometimes you're supposed to reach a father or mother who is in active substance abuse, and they are supposed to give their consent – ‘Yes my daughter is allowed to see a speech therapist’. Children whose parents had severe problems themselves thus received less support than the CHCNs deemed as needed, not only for recognised language delays but in general.

In sum, the interviews comprised rich data, enabling a comprehensive and profound analysis. The analysis provides substantial content to discuss the research question.

## 5 DISCUSSION

The study aimed to investigate the experience of CHCNs using language screening for 2.5‐year‐old children. The findings give useful insight into how the screening is used and experienced by CHCNs. The results challenge some of the ideas about the objectives of the screening and provide decision makers with information that can contribute to the development of new routines or adjustments of current routines for CHC.

### 5.1 The use of the instrument for language screening

The CHCNs had to make adjustments to the language screening procedures. It is noteworthy that the CHCNs clearly stated that they cannot follow the instructions, a fact that decreases the validity of the method and raises questions about which children are being identified in the screening. This finding might explain the lack of sensitivity and specificity detected in previous studies (Wallace et al., 2015). It also confirms the results from Jullien (2021) and Voigt and Accardo (2015), who question the function of screening outside of the experimental settings in validation studies.

Neurodevelopmental symptoms, most often features of autism, consumed a large part of the CHCN's attention during the language screening. However, the language screening is not evaluated for screening of neurodevelopmental symptoms, and the CHCNs did not use any other instruments to identify neurodevelopmental issues. The behaviours and features that the CHCNs described are not distinct features of autism and could just as well be symptoms of something else. This result indicates that the language screening is not being used only for identifying delayed language development, but also to identify autism. This result is interesting in light of the high number of autism spectrum disorders among children identified at 2.5 years in previous studies (Miniscalco et al., 2006). When features of autism are the focus of the CHCN's attention, it is reasonable to believe that symptoms of autism will be detected in some children. Identifying early signs of autism is an important objective of the CHC, but there are other aspects of children's health and development that can be of interest as well. The risk of being too specific in the early years is that it might obscure other possible explanations of observed symptoms. The surveillance of children's health should perhaps include risk factors related to psychological health other than autism. Risk factors, such as adverse life experiences, could be assessed in a valid way and included in an extended routine.

### 5.2 Negotiating with parents

The CHCNs sometimes had to rely on the parents' assessments, even when they were unsure of it. This finding is noteworthy in relation to previous research showing the insufficiency of parental assessments (Nayeb et al., 2021). The relationship with the parents is not a primary aim of the CHC programme, but it was clearly prioritised by the CHCNs in our study. One possible explanation is that the CHC national programme requires and supports long‐term relationships between CHCNs and parents. They first meet when a child is 1 week old, and they then follow a schedule with nine health visits during the child's first year and one more before the visit at age 2.5. The parents must give their consent for any referral; if they do not want to consent, the CHCN can contact social services for a report. It is understandable that a CHCN try to get parental support for their assessments and procedures. Nevertheless, the parents' influence on their children's care is crucial. When it comes to children exposed to maltreatment and other adverse life events, the CHCNs report that it is sometimes even harder to motivate the parents to follow interventions or to get a consent for referral. Children whose parents are more help‐seeking are more likely to get help than children whose parents are not. It is important to strive for health care for children in greatest need rather than just those with help‐seeking parents. The consequence of a system in which the child's health care is determined by their parents' capacity and motivation is that some children might not get the help they need.

### 5.3 Normativity in CHC

When an interpreter is needed during language screening a telephone interpreter is used. Our study indicates that interpretations of a 2.5‐year‐old child over the telephone can be considered unreliable. The purpose of the language screening, namely, to assess children's language development and communication, requires that the mode of interpretation be chosen with the needs of the patient and caregivers in mind (Ogunnaike et al., 2022; Rikshandboken, 2022). In the CHCNs statements it became obvious that the interpreter translated what the parents said, not verbal expressions from the child. If the mode of interpretation is inadequate for the task, identifying and helping bilingual children is compromised. The result is that for a part of the child population their results are not trustworthy, which could prevent their full participation in the health care system. A recent review highlights that these problems are frequent in CHC (Ogunnaike et al., 2022).

The challenges identified in working with families from ethnic minorities and with parents who do not speak Swedish may also relate to earlier studies reporting on how cultural, social and socioeconomic backgrounds influence values, beliefs and practices that impact parent–child interactions, the value of conversation, and in teaching language to children (Mesman et al., 2018; Rikshandboken, 2022). Who is talking to whom and the value of children's talk has been shown to be strongly based on cultural norms, possibly limiting the effects of language stimulation interventions (Mesman et al., 2018; Salameh & Nettelbladt, 2018). Intervention programmes encouraging parents to talk directly to their children are based on norms in western culture. In our study parents from ethnic minority cultures are encouraged to change their parenting to promote their children's language development. The advice to interact with children to stimulate language development, based as it is in western culture, can explain some of the challenges experienced in trying to convince parents with roots outside of western culture. Not one of the interviewed CHCNs questioned the norms of their own western culture concerning language development and child rearing. A related phenomenon was the CHCNs' opinion that children need to focus on learning one language properly, and that language delay may be explained by the use of two or more languages. This myth seems to be rather established although contradicted in the National CHC Program (Rikshandboken, 2022). This is in line with Stankova et al. (2021) stating that there is a gap between the cultural competence needed and practitioners' actual knowledge, a gap that practitioners are largely unaware of. This finding also relates to research showing that interventions tend to be homogenised and based on the dominant culture in contrast with existing diversity of children and families (Larson & Bradshaw, 2017).

### 5.4 Strengths and limitations

There are potential methodological limitations to the present study. The method does not permit generalisations of the results to other groups. Future research can reveal to what extent the experiences found in these themes are common or limited to the specific context. The first author who did the interviews, is a child psychologist within the CHC, which could potentially influence the statements in the interviews. The interview material was considered rich and nuanced, which implicates that the participants could express their thoughts without restraint.

## 6 CONCLUSIONS

Our findings indicate that a modified procedure for the language screening of children 2.5 years old is used in order to secure the child's cooperation, to adjust to practical circumstances, and to preserve an alliance with the parents. A consequence of this finding is that the validity of the screening can be called into question. In addition, the screening is used not only to identify children with language delays, which is the screening's purpose, but also to identify children with signs of autism. The question of validity is particularly apparent when it comes to children from families of ethnic minorities and children exposed to adverse life events. The findings underline the importance of further development of the routines for screening. Taking a broader view of the screening could help identify children at risk of psychological ill health and developmental delay and enable support and interventions for young children and their families.

## AUTHOR CONTRIBUTIONS

All authors participated in the conception and design of the study. The first author conducted all the interviews and transcription. All the authors participated in data analysis and reviewing the manuscript.

## FUNDING INFORMATION

This study was funded by the Region Värmland.

## CONFLICT OF INTEREST STATEMENT

No potential conflict of interest is reported by the authors.

## ETHICS STATEMENT

Ethical approved by the Swedish ethical board 2020‐09‐07, dnr 2020‐03537.

## CLINICAL TRIAL REGISTRATION NUMBER AND NAME OF TRIAL REGISTER

ID‐number: 274509.

## Data Availability

Data available on request from the authors. The data that support the findings of this study are available from the corresponding author upon reasonable.
